# Step-by-Step Clipping of Clustered Ipsilateral Anterior Circulation Aneurysms via an Extended Minipterional Approach

**DOI:** 10.7759/cureus.107070

**Published:** 2026-04-14

**Authors:** Alejandro Serrano-Rubio, Xavier Wong-Achi, Ambar Elizabeth Riley-Moguel, José Guillermo Flores-Vázquez, Edgar Nathal

**Affiliations:** 1 Vascular Neurosurgery, Instituto Nacional de Neurología y Neurocirugía Manuel Velasco Suárez, Mexico City, MEX; 2 Neurosurgery, Instituto Nacional de Neurología y Neurocirugía Manuel Velasco Suárez, Mexico City, MEX

**Keywords:** cerebrovascular surgery, microsurgery., minipterional approach, multiple brain aneurysms, surgical clipping, unruptured aneurysms

## Abstract

We describe the step-by-step microsurgical management of four right-sided incidental clustered ipsilateral anterior circulation aneurysms through a single extended minipterional approach. A 55-year-old woman with right-sided aneurysms involving the A1 segment, anterior choroidal artery, posterior communicating artery, and carotid terminus underwent clipping in a single-stage procedure. Her past medical history included two previously clipped aneurysms of the clinoidal segment of the right internal carotid artery (ICA). The patient was positioned supine with vertex-down tilt and contralateral head rotation to align the surgical corridor along the sphenoid ridge. A 4 × 5 cm craniotomy was performed following extradural sphenoid ridge drilling and anterior clinoidectomy to expose the distal dural ring and secure proximal internal carotid artery control. Sylvian fissure dissection from distal to proximal allowed cerebrospinal fluid drainage and brain relaxation. Aneurysms were clipped sequentially from proximal to distal: first the A1 aneurysm with preservation of perforators, followed by the posterior communicating artery aneurysm, the anterior choroidal artery aneurysm, and finally the carotid terminus aneurysm. Intraoperative fluorescein videoangiography confirmed aneurysm exclusion and patency of parent vessels and perforators. This stepwise proximal-to-distal strategy provided an unobstructed operative field, ensured vascular control, and allowed safe treatment of multiple lesions through a single surgical corridor.

## Introduction

The incidence of multiple intracranial aneurysms (MIAs) is approximately 7-35% of all intracranial aneurysms, and they are most commonly located in the anterior circulation [1-2]. As the number of aneurysms increases, the probability of rupture also doubles. According to the International Study of Unruptured Intracranial Aneurysms (ISUIA), the risk of rupture rises with a diameter greater than 7 mm [3-9]; however, in cases of MIAs, the risk of rupture of small aneurysms may be increased up to tenfold [2]. When one of the aneurysms ruptures, the primary goal is to secure the ruptured aneurysm while simultaneously treating the others without compromising surgical safety or patient outcomes [9, 10].

An in-depth understanding of the region's anatomy, combined with advanced microsurgical expertise, is essential for achieving successful surgical outcomes. Perforating arteries are of relevance in these cases. Numerous perforators are located posterior to the internal carotid artery (ICA) bifurcation. These originate from: 1) the anterior choroidal (AChoA) segment of the ICA, 2) the posterior communicating (PCom) segment, 3) the recurrent artery of Heubner (RAH), and 4) the medial and lateral lenticulostriate arteries (MLA and LLA, respectively) [7]. Proximal A1 aneurysms arise from the proximal segment (A1) of the anterior cerebral artery (ACA), often at the origin of perforators, and may be adherent to them. The perforators of the A1 segment can be divided into two groups: 1) the MLA and 2) the RAH [1].

The extended minipterional approach has emerged as a less invasive modification of the standard pterional craniotomy, designed to provide adequate exposure of the anterior circulation while minimizing soft tissue dissection and temporal muscle manipulation [5]. This approach is particularly useful in cases of multiple ipsilateral aneurysms, where a single surgical corridor may allow treatment of several lesions in one stage. Compared with the conventional pterional approach, the extended minipterional technique offers potential advantages such as reduced surgical trauma, shorter operative time, improved cosmetic outcomes, and decreased postoperative discomfort [5, 10-12]. At the same time, it preserves sufficient surgical freedom to achieve proximal and distal vascular control, which is critical in the management of complex aneurysms.

While endovascular techniques have become the first-line treatment for many intracranial aneurysms, microsurgical clipping remains an essential strategy in cases with complex morphology, unfavorable anatomy, or when endovascular therapy is not feasible or unavailable. In this context, minimally invasive microsurgical approaches such as the extended minipterional approach provide a valuable alternative, combining durability with reduced surgical morbidity [11].

However, this approach also has limitations, including a narrower operative corridor and increased technical demands, particularly in cases with high-positioned aneurysms or significant brain swelling. Therefore, careful patient selection and detailed preoperative planning are essential to ensure safe and effective outcomes.

## Technical report

We used a right extended minipterional approach [5]. The patient is positioned supine with the head fixed, tilted 20° vertex-down, and rotated approximately 30° to the contralateral side to provide a direct visual axis along the sphenoid ridge toward the anterior clinoid process (ACP). The frontozygomatic point and the pterion, representing the lateral end of the sphenoid ridge, are identified. Hair is strip-shaved 1 cm along the planned incision line. A semi-question mark-shaped skin incision is made, followed by a C-shaped inverted incision in the superficial temporalis fascia and muscle. The muscle is reflected using fishhooks until the orbital rim and pterion are exposed. A single burr hole is placed at the most caudal aspect of the exposure, and an oval craniotomy measuring approximately 4 × 5 cm is performed [4]. The patient positioning and preoperative incision planning can be visualized in the surgical video provided in the Appendix section.

**Figure 1:**
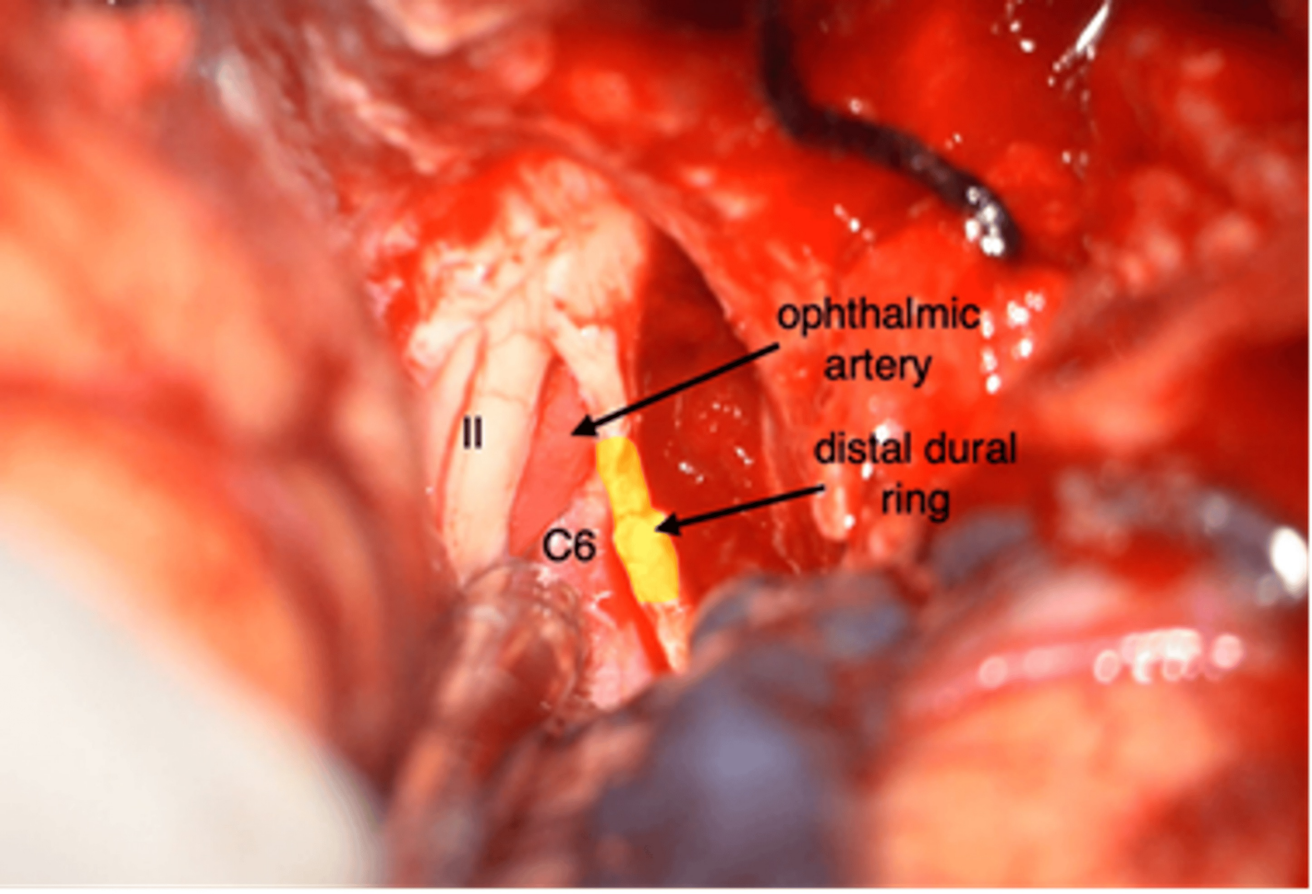
Ophthalmic artery and distal dural ring visualization This view shows the optic nerve (II), the ophthalmic segment of the ICA (C6), the ophthalmic artery, and the distal dural ring after the opening of the optic nerve sheath.

The sphenoid ridge and the lateral portion of the superior orbital fissure are resected using a cutting drill until the meningo-orbital band is exposed and cauterized. A 2-mm diamond drill and Kerrison rongeurs are then used to complete the extradural clinoidectomy. The dura is opened in a two-layer fashion. The optic nerve sheath is incised, allowing visualization of the ophthalmic artery and the distal dural ring, which is opened to expose the ICA, mobilize it, and obtain proximal vascular control, if necessary (Figure 1). In the event of venous bleeding at this stage, fibrin glue is applied to Mullan’s triangle and the carotid-oculomotor membrane.

**Figure 2:**
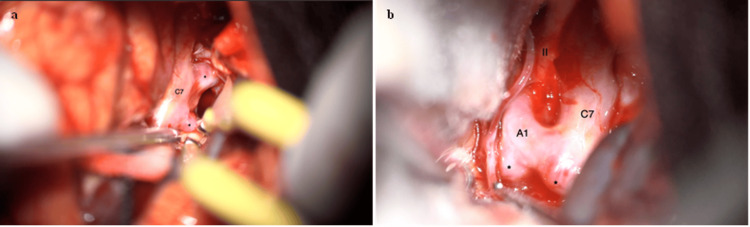
Intraoperative views of the right ICA (a) The communicating segment of the ICA (C7) is shown with aneurysms arising from the AChoA and PCom artery segments. (b) The optic nerve (II) and the A1 segment of the ACA are visualized. Asterisks indicate an aneurysm at the proximal A1 segment and another lesion at the ICA bifurcation.

Splitting of the Sylvian fissure proceeds from distal to proximal toward the C7 segment of the ICA, facilitating cerebrospinal fluid (CSF) drainage and brain relaxation. The carotid-oculomotor triangle is visualized, and the ICA is followed distally until the A1-M1 bifurcation is reached [6]. At this point, the four aneurysms are identified (Figure 2).

**Figure 3:**
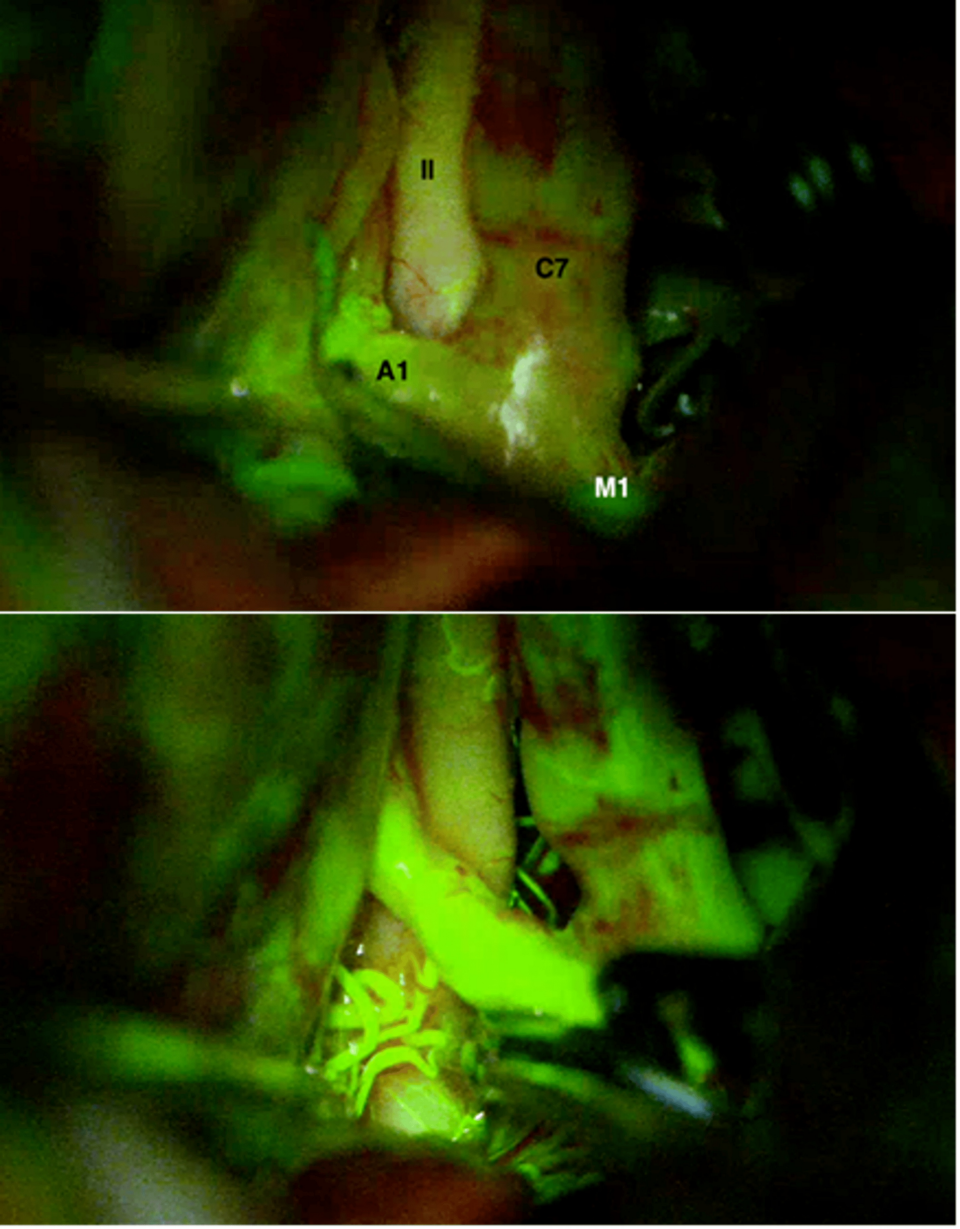
Intraoperative fluorescence angiography Fluorescence angiography is essential for evaluating distal flow in the main arteries and their branches, confirming both aneurysm occlusion and vessel patency.

**Figure 4:**
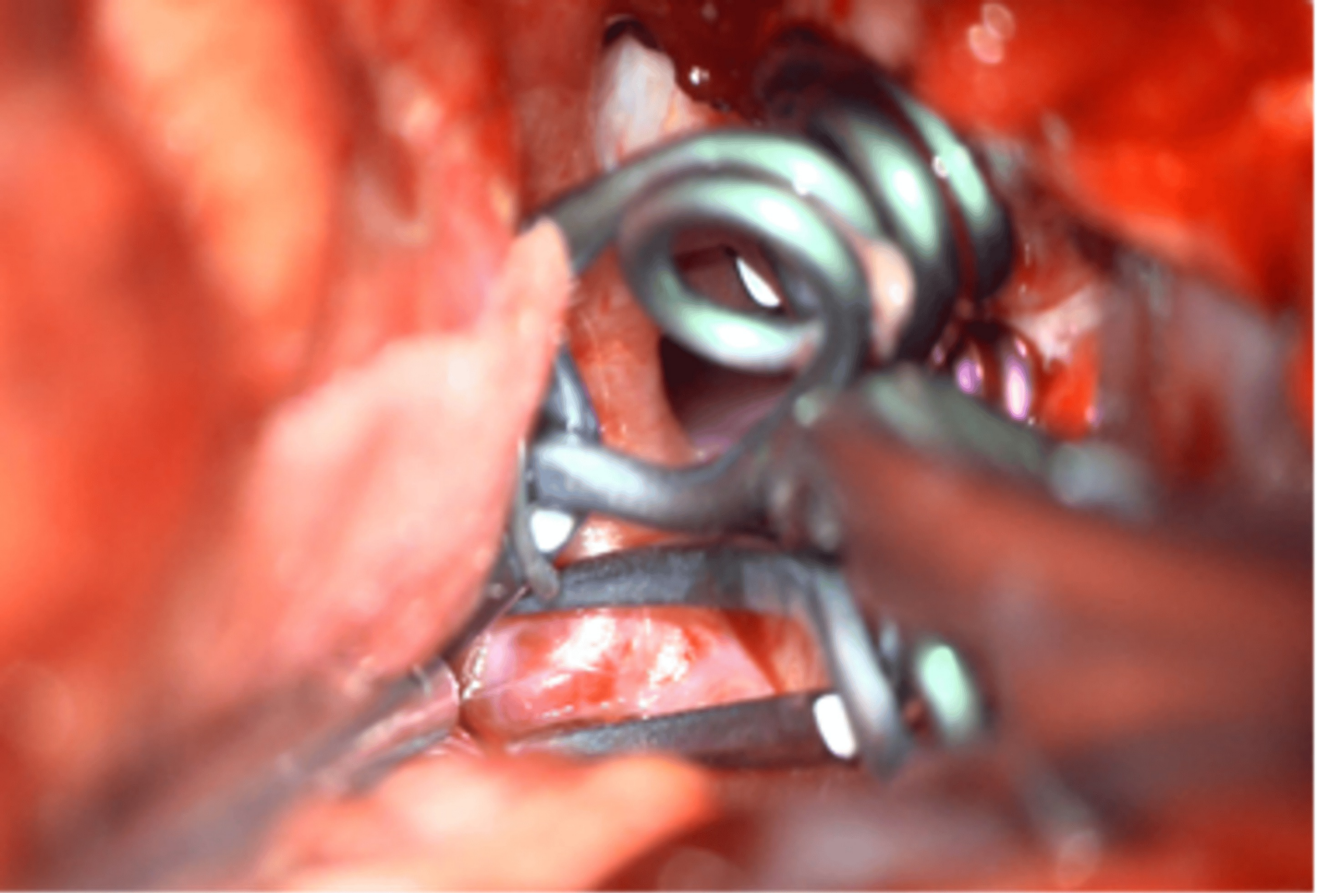
Final intraoperative view View after clipping three aneurysms sequentially from proximal to distal, followed by definitive clipping of the blister aneurysm. This strategy provided an unobstructed operative field, facilitating safe placement of all clips.

The dissection was initiated at the A1 segment aneurysm, with careful localization and visualization of the associated perforators. A semi-curved clip was applied to secure this aneurysm. The PCom artery segment aneurysm was then clipped using a straight 7-mm clip, followed by clipping of the AChoA segment aneurysm with a miniclip. Patency of the PCom and AChoA was confirmed intraoperatively using fluorescein videoangiography, and patency of the A1 segment perforators was also verified (Figure 3). A 5-mm semi-curved clip was used to secure the carotid terminus aneurysm (Figure 4).

After clipping, hemostasis was achieved, and a hemostatic sponge was placed within the Sylvian fissure. A watertight dural closure was performed to prevent cerebrospinal fluid leakage. The bone flap was fixed with silk sutures, and the temporal fascia and muscle were closed using 2-0 absorbable sutures. The complete procedure can be observed in the surgical video included in the Appendix section.

**Figure 5:**
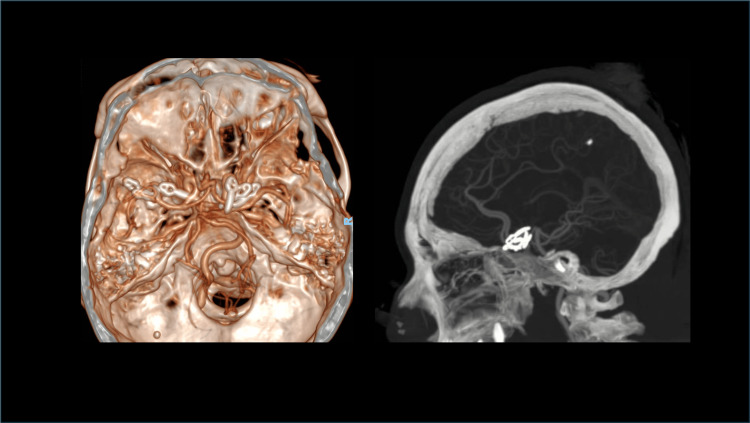
Three-dimensional reconstruction from postoperative CT angiography Postoperative imaging demonstrating sequential proximal-to-distal clipping of three aneurysms, followed by definitive clipping of the blister aneurysm. This stepwise strategy provided an unobstructed operative field and facilitated safe and accurate clip placement.

All four aneurysms were successfully clipped. The postoperative course was uneventful, with no new neurological deficits. Postoperative imaging confirmed complete exclusion of all aneurysms without evidence of residual filling or vascular compromise (Figure 5). The patient was discharged in stable condition and remained neurologically intact at follow-up.

## Discussion

Intracranial aneurysms are defined as focal dilations arising at structurally weak points within the cerebral arterial circulation and are present in approximately 3% to 5% of the general population. Their rupture is the leading cause of spontaneous subarachnoid hemorrhage, accounting for nearly 85% of cases. Multiple aneurysms are identified in up to 30% of all intracranial aneurysm cases [12-15].

Multiple intracranial aneurysms, whether incidental or ruptured, can be treated with microsurgical clipping in one or two stages, depending on their location and complexity. Although each case must be individualized, surgical planning should include a detailed clipping strategy, selection of appropriate clips, and thorough assessment of the relevant local anatomy. This is the technique employed at our institution, with favorable clinical results. Previous studies have reported complete occlusion rates exceeding 90% for microsurgical clipping of anterior circulation aneurysms, with lower recurrence and retreatment rates compared to endovascular techniques, although at the cost of higher initial invasiveness [14-15].

Managing MIAs is challenging, primarily due to two factors: selecting the optimal surgical approach and accurately identifying the ruptured aneurysm. When one aneurysm ruptures in the context of multiple lesions, the primary objective is to secure the ruptured aneurysm while safely clipping the remaining aneurysms without compromising the patient’s outcome [13-15].

How to avoid complications?

To reduce complications when using the extended minipterional approach, several technical points should be emphasized. Proper head positioning should align the surgical corridor with the sphenoid ridge and anterior clinoid process while minimizing brain retraction. Tailored extradural drilling of the sphenoid ridge and anterior clinoid process, as well as wide opening of the basal cisterns, combined with distal-to-proximal Sylvian fissure dissection, facilitates cerebrospinal fluid drainage and improves surgical exposure through a limited craniotomy.

In cases of clustered aneurysms, stepwise dissection with early identification of perforators is critical. Sequential proximal-to-distal clipping enhances visualization and reduces operative crowding, particularly in regions involving the posterior communicating artery, anterior choroidal artery, and A1 segment, where perforator preservation is paramount. Intraoperative fluorescence angiography should be performed after clip placement to confirm aneurysm occlusion, verify parent vessel and perforator patency, and detect clip-related stenosis requiring immediate repositioning. This technique is a safe and reproducible adjunct that improves intraoperative decision-making; although it does not replace digital subtraction angiography, it provides real-time information that may help prevent ischemic complications [13-15].

Understanding regional and local anatomy is essential, as it helps prevent complications and guides surgical planning. A meticulous review of preoperative imaging studies - such as CT, CT angiography, and digital subtraction angiography - is critical for determining the optimal approach in each case. Particular attention should be given to preserving perforating arteries to ensure procedural safety.

Specific information for the patient

First, although this case involves multiple aneurysms, the minipterional approach offers excellent cosmetic results, and its less invasive dissection of the temporal muscle contributes to improved functional outcomes. Second, occlusion of the recurrent artery of Heubner or other perforating vessels can result in hemiparesis, hemianesthesia, or dysarthria, with additional symptoms depending on the main artery involved. While some of these deficits may improve over time, others may persist. Third, meticulous reconstruction of the surgical area is essential to prevent cerebrospinal fluid leakage.

Key points

Successful management of multiple intracranial aneurysms requires precise mastery of microsurgical anatomy and a thorough preoperative analysis of all imaging studies to guide surgical planning. Small skin and muscle incisions contribute to improved cosmetic and functional outcomes while maintaining adequate exposure. Accurate identification of key anatomical structures, particularly perforating vessels, is critical to avoid neurological complications. A proximal-to-distal clipping sequence helps maintain a safe and unobstructed operative field, facilitating proper clip placement. Securing both proximal and distal vascular control before definitive clipping enhances procedural safety. Hemostatic agents or fibrin glue may be necessary when working near the cavernous sinus to manage venous bleeding effectively. The decision to perform a single-stage or staged procedure should be individualized based on aneurysm location and complexity. Smaller surgical approaches may reduce operative time and intraoperative bleeding when properly planned. Finally, in cases involving ruptured aneurysms, accurate identification of the ruptured lesion is essential to determine the initial surgical strategy and optimize patient outcomes.

## Conclusions

Microsurgical clipping of multiple ipsilateral anterior circulation incidental intracranial aneurysms through a single extended minipterional approach is a safe and effective strategy in carefully selected patients when performed with meticulous surgical planning and microsurgical technique. This approach provides adequate exposure, proximal vascular control, and the ability to treat multiple lesions through a single surgical corridor.

A stepwise proximal-to-distal clipping sequence, supported by intraoperative fluorescence angiography, facilitates confirmation of aneurysm occlusion and preservation of perforator vessels, contributing to procedural safety. Although technically demanding, this technique represents a valuable microsurgical option for the management of complex cases of multiple unruptured intracranial aneurysms.

## References

[1] Boutarbouch M, Dokponou YC, Bankole ND, El Ouahabi A, El Khamlichi A (2023). Evaluation of unruptured aneurysm scoring systems and ratios in subarachnoid hemorrhage patients with multiple intracranial aneurysms. Surg Neurol Int.

[2] Chen J, Tong X, Feng X (2021). Management of unruptured small multiple intracranial aneurysms in China: a comparative effectiveness analysis based on real-world data. Front Neurol.

[3] Juvela S (2021). Outcome of patients with multiple intracranial aneurysms after subarachnoid hemorrhage and future risk of rupture of unruptured aneurysm. J Clin Med.

[4] Neyazi B, Swiatek VM, Skalej M (2020). Rupture risk assessment for multiple intracranial aneurysms: why there is no need for dozens of clinical, morphological and hemodynamic parameters. Ther Adv Neurol Disord.

[5] Qian W, Chen Y, Zhu Q, Chen A, Lan Q (2024). Microsurgical clipping of multiple intracranial aneurysms via the keyhole approach. World Neurosurg.

[6] Rosi Junior J, Gomes Dos Santos A, da Silva SA (2021). Multiple and mirror intracranial aneurysms: study of prevalence and associated risk factors. Br J Neurosurg.

[7] Sato H, Kamide T, Kikkawa Y (2021). Clinical characteristics of ruptured intracranial aneurysm in patients with multiple intracranial aneurysms. World Neurosurg.

[8] Seong J, Kim J, Lee S, Kim B (2024). Two consecutive ruptured intracranial aneurysm in patient with multiple intracranial aneurysms. J Cerebrovasc Endovasc Neurosurg.

[9] Tong X, Feng X, Peng F (2023). Rupture discrimination of multiple small (< 7 mm) intracranial aneurysms based on machine learning-based cluster analysis. BMC Neurol.

[10] Xin WQ, Sun PJ, Li F (2020). Risk factors involved in the formation of multiple intracranial aneurysms. Clin Neurol Neurosurg.

[11] Kendirlioglu BC, Sulaimanov U, Erginoglu U (2026). Comparative outcomes of microsurgical and endovascular treatment for ruptured and unruptured anterior communicating artery aneurysms. Neurosurg Rev.

[12] Caplan JM, Papadimitriou K, Yang W (2014). The minipterional craniotomy for anterior circulation aneurysms: initial experience with 72 patients. Neurosurgery.

[13] Nathal E, Serrano-Rubio A, Xochipa-Ruiz KE (2026). Application of intraoperative fluorescent imaging techniques in vascular neurosurgical procedures. Egypt J Neurosurg.

[14] Al-Jehani H, Mousa AH (2025). Microsurgical clipping versus endovascular coiling for the treatment of anterior circulation intracranial aneurysms: a systematic review. Discov Med.

[15] Gómez-Amador JL, Villanueva-Castro E, Nathal-Vera E (2025). Clinical and surgical outcomes of bilateral intracranial aneurysms clipped through a single craniotomy: a retrospective comparative cohort study. Neurosurg Focus.

